# Complicated Superior Mesenteric Artery Syndrome

**DOI:** 10.7759/cureus.96723

**Published:** 2025-11-12

**Authors:** Raja Alqandouz, Afra A mohamed, Saba Sharafuddin, Yaman AlAhmad, Jignesh Trivedi, Pradeep Radhakrishnan, Vamanjore A Naushad

**Affiliations:** 1 Internal Medicine, Hamad General Hospital, Doha, QAT; 2 Medicine, Hamad Medical Corporation, Doha, QAT; 3 Clinical Imaging Department, Hamad Medical Corporation, Doha, QAT; 4 Medicine, Hamad General Hospital, Doha, QAT; 5 Clinical Department, College of Medicine, QU Health, Qatar University, Doha, QAT; 6 Clinical Medicine, Weill Cornell Medicine, Doha, QAT

**Keywords:** duodenum, obstructive symptoms, scleroderma, sma syndrome, superior mesenteric artery syndrome

## Abstract

Superior mesenteric artery syndrome (SMA) is a rare condition more commonly seen in young, thin females. In SMA syndrome, the duodenum is compressed between the SMA and the aorta, and the patient presents with obstructive symptoms. Here, we present a case of complicated SMA syndrome in a young patient with limited scleroderma. She presented with abdominal pain, early satiety, and significant weight loss. Her condition was further complicated by the development of emphysema. This case illustrates the diagnostic challenge and complexity of managing SMA syndrome in the context of connective tissue disorders with multisystemic manifestations.

## Introduction

Superior mesenteric artery (SMA) syndrome, also known as cast syndrome, Wilkie syndrome, or aortomesenteric duodenal compression syndrome, is uncommon and was first described by Rokitansky in 1842. The incidence of SMA syndrome ranges from 0.013-0.3% to 0.78% [1]. Three factors have been identified as causes: increased extra- or intra-abdominal pressure, decreased body weight, and elevated mesenteric tension [2]. SMA syndrome is associated with autoimmune diseases such as systemic lupus erythematosus (SLE) [3]. Cases of generalized scleroderma with SMA syndrome have been rarely reported; however, limited scleroderma with SMA syndrome is exceedingly uncommon. We report a case of complicated SMA syndrome in a female patient with limited scleroderma.

## Case presentation

A 17-year-old female was admitted to the hospital with complaints of nausea, vomiting, and abdominal pain lasting four days. The abdominal pain was primarily localized to the epigastric region, and vomiting occurred mostly after meals. She reported an inability to tolerate oral intake and had lost 10 kg in the two weeks before admission. She denied fever, hematemesis, urinary or bowel complaints, or other systemic symptoms. Her medical history included limited scleroderma for six years, without lung or gastrointestinal involvement, adequately controlled with a daily oral dose of 10 mg methotrexate. There were no skin manifestations at the time of her admission.

Initial blood tests showed a normal white blood cell count, renal and liver function parameters, and pancreatic enzyme levels (Table 1).

**Table 1:** Basic laboratory findings CRP: C-reactive protein; ANA: antinuclear antibody; CTD: connective tissue disease; ALT: alanine aminotransferase; AST: aspartate aminotransferase; Alk phos: alkaline phosphatase; Anti SCL 70: anti-scleroderma 70 antibody

Tests	Upon Admission	D3	D5	Normal Reference Range
White blood cell	4.7	5.6	10	(4 – 10 × 103/ UL)
Hemoglobin	12.2	12.5	11.8	(12–15 gm/dL)
Platelet	264	289	295	(150-400 × 103 / µL)
CRP	0.8	2.1	13.4	(0.0-5.0 mg/L)
Creatinine	44	43	47	(38 -97 umol/L)
Urea	0.7	2.6	2.2	(2.1 - 8.1 mmol/L)
Potassium	3.6	3	3.2	(3.3-5.3 mmol/L)
ALT	10	9	11	(0-30 U/L)
AST	15	15	16	(0-31 U/L)
Alk Phos	85	69	69	(33.5 -129 U/L)
Bilirubin Total	21	17	12	(3.5 - 24 umol/L)
Albumin	36	35	37	(35-50 gm/L)
ANA	Negative			
ANA CTD	Positive			
Anti SCL 70	Negative			
Anti-Centromere	Negative			

Abdominal ultrasound was reported as normal. Based on the clinical symptoms, signs, and initial test results, a provisional diagnosis of gastritis was made, and she was treated with parenteral proton pump inhibitor (PPI), antiemetics, and intravenous fluids.

**Figure 1:**
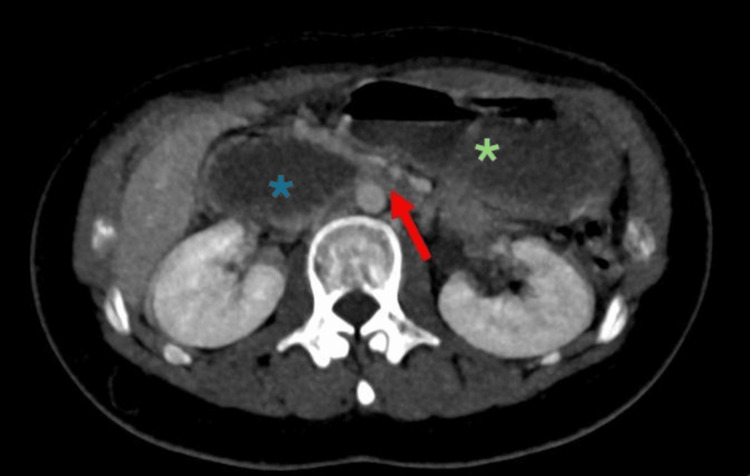
Axial abdomen slice shows marked dilatation of the stomach (green asterixis), first (D1), second (D2), and proximal third (D3) parts of duodenum (blue asterixis) with transition point at the aortomesenteric region (red arrow) (between the superior mesenteric artery (SMA) and abdominal aorta).

**Figure 2:**
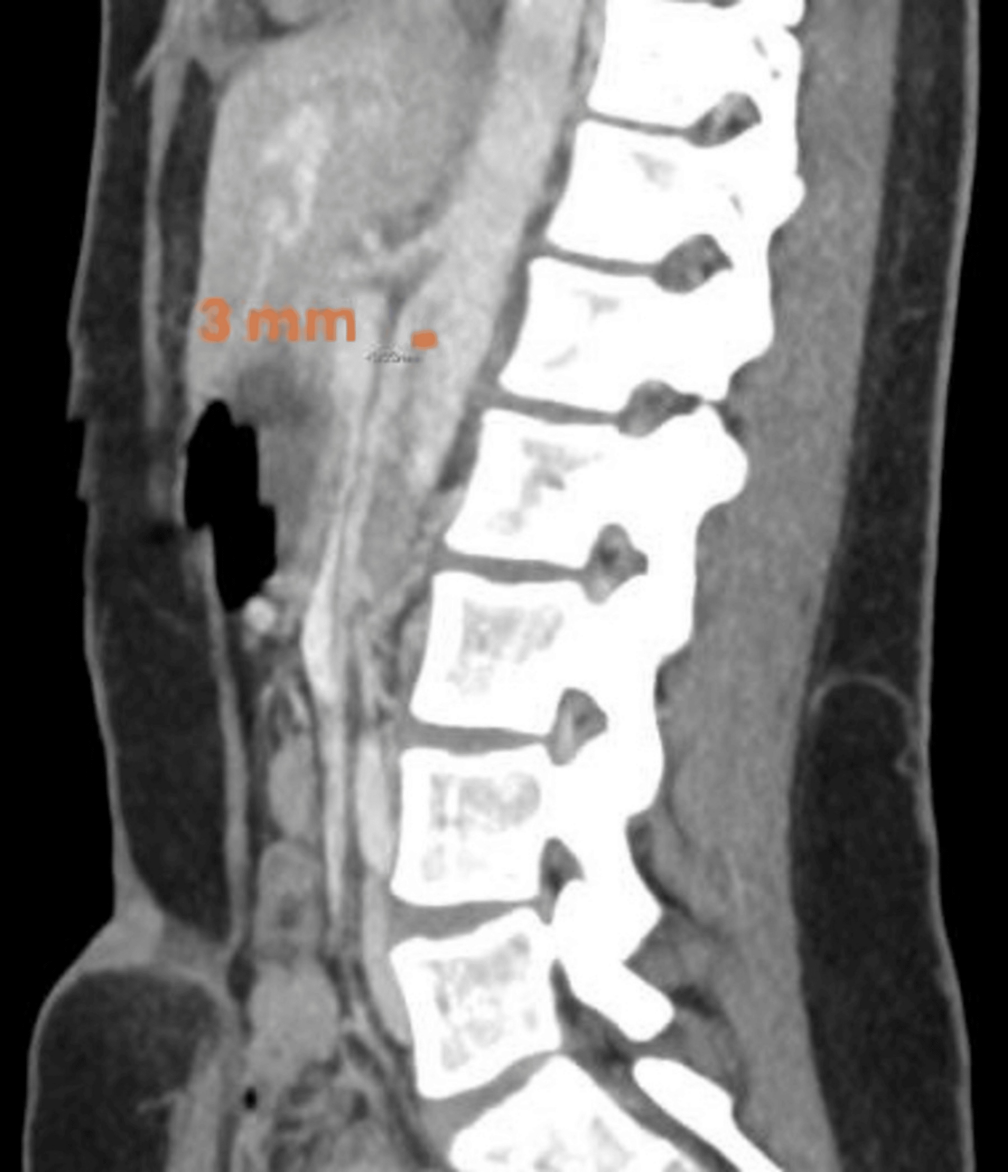
Sagittal CT abdomen slice used to evaluate for aortomesenteric distance measures 3 mm (orange bar), in keeping with superior mesenteric artery syndrome.

As her pain persisted, a CT scan of the abdomen was performed, which revealed dilatation of the first and second parts of the duodenum with compression of the third part, consistent with SMA syndrome (Figures 1-2).

Esophagogastroduodenoscopy (EGD) and a barium meal study were normal. A multidisciplinary team (MDT) meeting was convened, including upper gastrointestinal (UGI) surgery, internal medicine, rheumatology, anesthesia, pain management, and nutrition, to discuss the most appropriate management plan.

From a surgical perspective, after reviewing the clinical presentation, CT findings, EGD, and barium swallow results, no correlation was observed between the imaging findings and her symptoms. They recommended continuing medical management without surgical intervention, with follow-up.

Although most of her scleroderma manifestations were confined to the skin (primarily morphea), and this type is unlikely to affect internal organs, the possibility of a rare association between scleroderma and SMA syndrome was considered. Methotrexate therapy was continued as advised by rheumatology.

She was treated with PPI, prokinetic medications, and appropriate pain management. Nutritional advice included small, frequent meals every two to three hours, a high-protein, high-calorie pureed diet, and nutritional supplements.

**Figure 3:**
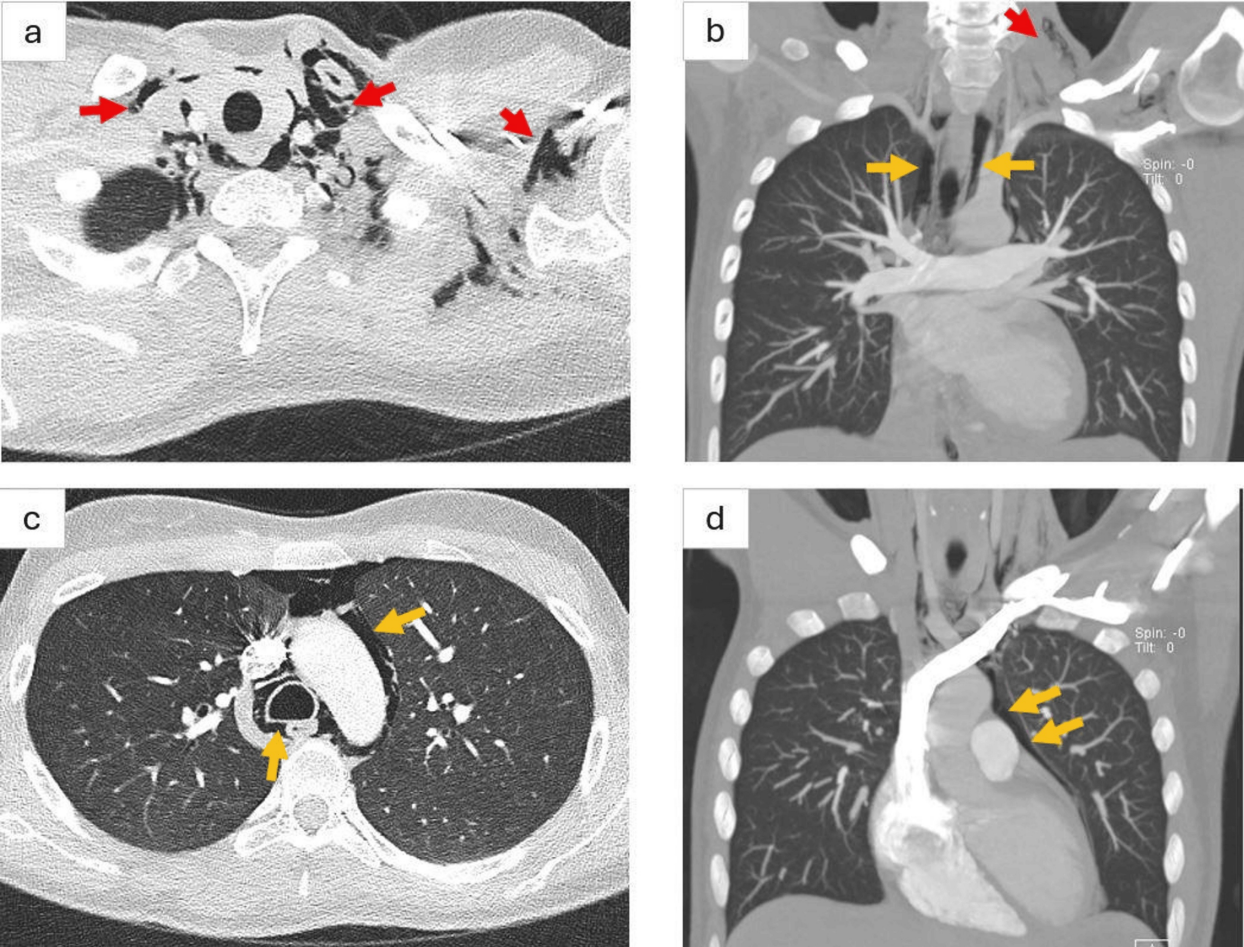
Intravenously enhanced computed tomography pulmonary angiogram. Selected images of axial (a, c) and coronal (b, d) planes of intravenously enhanced computed tomography pulmonary angiogram (lung window) revealed significant subcutaneous and inter-fascial air density seen on both sides of the lower neck (red arrows) and the left axilla. Further air density is seen at the mediastinum and along the azygoesophageal recess (orange arrows). Features in keeping with surgical emphysema and pneumomediastinum.

On the 13th day of admission, she developed central chest pain without other new symptoms. An urgent CT pulmonary angiogram was performed to rule out pulmonary embolism, which was negative. However, extensive pneumomediastinum was observed (Figures 3a-3d). UGI surgeons diagnosed Boerhaave syndrome. An urgent EGD was performed with the possibility of stenting, but no perforation was found. The UGI team opted for medical management of the pneumomediastinum with parenteral antibiotics and antifungal medications.

She was managed conservatively as per the multidisciplinary team plan, and her symptoms showed significant improvement with medical treatment. She was discharged after 20 days of hospitalization. At a one-month follow-up in the outpatient department, she was doing well and reported an improvement in symptoms along with a weight gain of 4 kg.

## Discussion

We present a case of SMA syndrome in a patient with previously diagnosed limited scleroderma, who presented with abdominal pain, repeated vomiting, and significant weight loss. Investigations confirmed SMA syndrome, and she later developed pneumomediastinum as a complication due to repeated vomiting, leading to esophageal rupture.

SMA syndrome occurs more frequently in females than males, especially young, thin females, though it can develop at any age. A review by Oka et al. reported a median age of 23 years (interquartile range 16-39), with a female-to-male ratio of 3:2 [1]. Radiographic studies estimate the incidence in the general population at 0.013% to 0.78% [2].

SMA syndrome develops when the third portion of the duodenum is compressed between the superior mesenteric artery anteriorly and the aorta posteriorly. This usually results from loss of the fat pad between the duodenum and the SMA, leading to narrowing of the aortomesenteric angle and reduced distance. Normally, the aortomesenteric angle ranges from 28° to 65°, and the distance ranges from 10 to 34 mm. In SMA syndrome, the angle decreases to 6°-22°, and the distance to 2-8 mm [4,5].

The underlying causes of SMA syndrome may be congenital or acquired. Congenital causes include spinal deformities, abnormal insertion of the ligament of Treitz, and a low origin of the SMA [1]. Acquired causes include rapid weight loss due to malabsorption syndromes, eating disorders, bariatric surgery, malignancy, or chronic infections such as HIV and tuberculosis [6]. Autoimmune diseases, including SLE and Graves’ disease, have also been reported as precipitating factors. SMA syndrome can also follow severe burns or spinal cord injury.

The clinical presentation is often nonspecific and mimics other causes of upper gastrointestinal obstruction [7]. Symptoms may include bloating, early satiety, postprandial discomfort, vomiting, epigastric pain, and weight loss. Epigastric pain typically improves in the lateral decubitus position and worsens when prone [8]. Many patients are initially treated for dyspepsia or gastritis.

In the present case, the likely mechanism of duodenal compression was rapid weight loss leading to reduced mesenteric fat and narrowing of the aortomesenteric angle. Unlike typical SMA syndrome, postural changes did not alleviate her pain.

SMA syndrome can be classified as acute or chronic depending on the cause and duration of symptoms [2,9]. Acute SMA syndrome usually follows trauma, surgery, or burns, and presents with recurrent epigastric pain, bilious vomiting, and weight loss. Chronic SMA syndrome is associated with conditions that cause low body mass index, such as malabsorption syndromes or scleroderma, and presents with indigestion, nausea, vomiting, and intermittent biliary vomiting over months to years [2]. In our patient, symptoms were of short duration: abdominal pain and vomiting for four days and weight loss for two weeks before admission.

Other clinical features of SMA syndrome often result from complications of frequent vomiting rather than duodenal compression itself. These may include dehydration, oliguria, and electrolyte abnormalities such as metabolic alkalosis and hypokalemia. Our patient was persistently hypokalemic during hospitalization. If unrecognized, SMA syndrome can lead to life-threatening complications, including hypovolemic shock, aspiration pneumonia, or sudden death. Sudden death may result from arrhythmia due to electrolyte imbalance, inferior vena cava compression by a dilated duodenum, or severe respiratory depression from alkalosis and increased abdominal pressure.

The most frequent complication is gastric mucosal injury from retained or refluxed gastric and bile acids with increased intraluminal pressure. The incidence of mucosal injury ranges from 25% to 59%. Chronic mucosal injury may progress to emphysema, necrosis, or pneumoperitoneum. Increased pressure in the second part of the duodenum may also obstruct pancreatic outflow, leading to acute pancreatitis [1,8].

SMA syndrome is often an incidental finding during evaluation of abdominal pain and vomiting. Without high clinical suspicion, the diagnosis is frequently missed or delayed. Diagnosis relies on characteristic symptoms together with imaging evidence of duodenal obstruction [1].

Plain abdominal X-rays may show duodenal dilatation and gastric gas. Barium studies may demonstrate dilation of the first and second portions of the duodenum, with or without gastric distension, acute angulation of the third portion, retrograde passage of contrast proximal to obstruction, marked delay in gastric emptying (4-6 h), and resolution of obstruction when the patient is positioned prone or in the lateral decubitus posture [1]. However, a normal X-ray or barium study does not exclude SMA syndrome, as in our case.

Historically, the aortomesenteric angle was measured by Doppler ultrasound, but contrast-enhanced CT of the abdomen and pelvis is now the gold standard [6]. CT with multiplanar reconstruction provides accurate vascular anatomy and allows direct measurement of the aortomesenteric angle and distance. An angle less than 22° has a sensitivity of 42.8% and specificity of 100%, while a distance less than 8 mm has both 100% sensitivity and specificity, making it more reliable [10].

The mainstay of treatment for SMA syndrome is conservative management, which includes bowel decompression, adequate hydration, and nutritional support. The primary goal of nutritional therapy is to relieve duodenal compression by promoting weight gain and restoring the mesenteric fat pad. Nutritional support often consists of small, frequent, high-calorie meals to increase mesenteric fat and relieve obstruction [7,11,12]. If oral intake is insufficient, enteral feeding through a percutaneous jejunostomy or nasojejunal tube may be required.

Anticoagulation may be considered to prevent thrombosis due to turbulence or stasis in the SMA or its branches. Prokinetic agents such as erythromycin or metoclopramide can improve gastric emptying and motility. Positional therapy, such as lying on the left side or prone, may also help alleviate compression. There is no predefined duration for conservative management, as gastrointestinal recovery may be delayed, and duodenal atony may persist even after decompression [7,12].

Surgical intervention is rarely required. When indicated, procedures include duodenojejunostomy, gastrojejunostomy, division of the ligament of Treitz, or vascular repair. Indications for surgery include failure of conservative therapy, progressive weight loss, persistent duodenal dilatation with stasis, or complications such as pancreatitis and peptic ulcers due to bile reflux. Patient preference for definitive surgical treatment or life-threatening complications such as bleeding, ischemia, or perforation may also necessitate surgery [7,12].

The prognosis is generally favorable if SMA syndrome is diagnosed early and treated appropriately. However, severe or undiagnosed cases can lead to potentially fatal complications such as aspiration pneumonia, hypocalcemia, esophageal injury, metabolic alkalosis, upper gastrointestinal bleeding, hypovolemic shock, and sudden death, though the exact mechanism of sudden death remains unclear [1,7,9].

In our patient, repeated vomiting led to pneumomediastinum and Boerhaave syndrome, which was successfully managed conservatively.

## Conclusions

Clinical manifestations of SMA syndrome are nonspecific and can mimic more common conditions, leading to delayed or missed diagnosis. Although SMA syndrome is typically benign, if symptoms are severe and management is inadequate, serious complications and death can occur. A high index of suspicion is therefore essential, particularly in patients with significant weight loss from various causes.

## References

[1] Oka A, Awoniyi M, Hasegawa N, Yoshida Y, Tobita H, Ishimura N, Ishihara S (2023). Superior mesenteric artery syndrome: Diagnosis and management. World J Clin Cases.

[2] Ahn TY, Han JB, Bae JY, Woo SH (2023). Superior mesenteric artery syndrome in a patient with fibrodysplasia ossificans progressiva. Bone Rep.

[3] Bradley IC, Trivedi B, Brockman MJ, Hassan M, Sotelo J, Okopie T, Dihowm F (2023). Superior mesenteric artery syndrome in systemic lupus erythematosus. Cureus.

[4] Bozzola E, Irrera M, Cirillo F (2024). Superior mesenteric artery syndrome in anorexia nervosa: A case report and a systematic revision of the literature. Nutrients.

[5] Waheed KB, Shah WJ, Jamal A (2021). Superior mesenteric artery syndrome: An often overlooked cause of abdominal pain!. Saudi Med J.

[6] Vakil R, Zingade AP, Baviskar M (2024). Superior mesenteric artery syndrome managed laparoscopically: A case report. J Med Case Rep.

[7] Soqia J, Janoud O, Soukia A, Saadoun R, Mousa K (2024). Challenges and pitfalls in diagnosing superior mesenteric artery syndrome: A case report. SAGE Open Med Case Rep.

[8] Ismail S, Hasan M, Aljasem M (2024). Superior mesenteric artery syndrome misdiagnosed and preceded by Helicobacter pylori-induced gastritis: A rare diagnosis with misleading features. Ann Med Surg (Lond).

[9] Bernotavičius G, Saniukas K, Karmonaitė I, Zagorskis R (2016). Superior mesenteric artery syndrome. Acta Med Litu.

[10] El Yousfi Z, Halfi IM, El Houss S, Allali N, El Haddad S, Chat L (2024). Superior mesenteric artery syndrome: A misdiagnosed disorder. Glob Pediatr Health.

[11] Lao BB, Pitts AM, Thomas A, Camacho SM (2023). Superior mesenteric artery syndrome and nutcracker syndrome as the presentation of Crohn's disease in a young patient: A case report and review of literature. JPGN Rep.

[12] Belo Guerra JDM, Mendonça Neto JS, Gomes de Freitas SR, Nogueira KD, Miranda de Souza LK (2023). Superior mesenteric artery syndrome: A systematic review. Int J for Innovat Educ Res.

